# An unfavorable intrauterine environment may determine renal functional capacity in adulthood: a meta-analysis

**DOI:** 10.6061/clinics/2018/e401

**Published:** 2018-10-05

**Authors:** Janaína Campos Senra, Mariana Azevedo Carvalho, Agatha Sacramento Rodrigues, Vera Lúcia Jornada Krebs, Maria Augusta Bento Cicaroni Gibelli, Rossana Pulcineli Vieira Francisco, Lisandra Stein Bernardes

**Affiliations:** I Departamento de Obstetricia e Ginecologia, Faculdade de Medicina FMUSP, Universidade de Sao Paulo, Sao Paulo, SP, BR; II Unidade Neonatal, Departamento de Pediatria Faculdade de Medicina FMUSP, Universidade de Sao Paulo Sao Paulo, SP, BR

**Keywords:** Fetal Growth Retardation, Kidney Disease, Systematic Review, Meta-Analysis

## Abstract

Since studies show that an unfavorable environment during intrauterine development predisposes individuals to several diseases in adulthood, our objective is to assess the relation between fetal growth restriction and chronic renal disease in adults. We searched four different electronic databases through November 2017: CENTRAL, EMBASE, LILACS and MEDLINE. We selected studies with longitudinal or transversal designs associating kidney function in adulthood with low birth weight. Two reviewers evaluated the inclusion criteria and the risk of bias and extracted data from the included papers. Thirteen studies were selected for the systematic review and meta-analysis. We observed increased risks of presenting end-stage renal disease (risk ratio 1.31, 95% confidence interval: 1.17, 1.47), a lower glomerular filtration rate (ml/min) (mean difference 7.14; 95% confidence interval: -12.12, -2.16), microalbuminuria (risk ratio 1.40; 95% confidence interval: 1.28, 1.52) and a small increase in the albumin/creatinine ratio (mean difference 0.46; 95% confidence interval: 0.03, 0.90) in the low birth weight patients, compared with control group. These findings suggest that low birth weight is associated with renal dysfunction in adults.

## INTRODUCTION

The number of nephrons an individual has is determined before birth, and more than half of these develop in the last three months of pregnancy, up until 36 weeks 1,2. Growth restriction is a condition in which a fetus fails to grow to its genetically determined potential *in utero* 3, and it is related to a reduction of more than one-third of the nephron population, which is not rectified after birth 4,5.

A reduced nephron number is critical in the progression toward chronic renal failure, and the remaining nephrons exhibit increased glomerular filtration, hyperperfusion, compensatory hypertrophy, microalbuminuria, arterial hypertension, glomerulosclerosis, overt proteinuria, and progressive fibrosis 6,7. Most studies have focused on fetal exposure to glucocorticoids in stressful situations as a plausible mechanism for reductions in the number of nephrons 8. This additional intrauterine exposure to glucocorticoids is critical in the determination of kidney diseases and hypertension; it is a factor in growth inhibition 9 and a frequent source of physiological and structural alterations associated with blood pressure adjustments, such as a reduction in nephron number 10, activation of the renin angiotensin system 11,12 and changes in the hypothalamic-pituitary-adrenal axis 13. However, although some studies show an influence of low birth weight (LBW) on kidney function later in life, no *consensus* is available regarding the factors that increase the risk, if any exist. Low birth weight may increase the probability of developing decreased renal function in adulthood. The investigations to date have been heterogeneous with respect to age, associated diseases, population, and definitions of outcomes; few studies have tried to synthesize all available data, but the populations evaluated were heterogeneous and were not restricted to adulthood because children and individuals with a history of extreme prematurity were also considered 14-16. Therefore, information must be collected to investigate the influence of intrauterine growth restriction (IUGR) on the development of kidney disease in adulthood.

Therefore, our objective was to evaluate the current literature on the influence of IUGR on renal disease in adulthood.

## METHODS

This study was conducted following the recommendations of the Cochrane Handbook for Systematic Reviews of Interventions, The Cochrane Collaboration, Version 5.1.0 17.

The protocol for this review was registered at PROSPERO (CRD42017040019).

The electronic search was conducted through November of 2017 using the following electronic databases: Cochrane Central Register of Controlled Trials (CENTRAL), Latin American and Caribbean Health Sciences (LILACS), Medical Literature Analysis and Retrieval System Online (MEDLINE), and EMBASE. The terms used in the Pubmed/MEDLINE search were (“Kidney Diseases” [Mesh]) AND “Fetal Growth Retardation” [Mesh] with no filters for species, sex or languages. The same terms were applied in EMBASE and CENTRAL, but with their extended trees. In LILACS, the search strategy was (“Nefropatias” OR “Kidney Diseases” OR “Enfermedades Renales”) AND (“Retardo do Crescimento Fetal” OR “Fetal Growth Retardation” OR “Retardo del Crecimiento Fetal”). Initially, no filters were applied for the study design, date, or language. The authors also conducted manual searches in the included studies and reviewed the references available on the subject to identify other potentially eligible articles.

The inclusion criteria were as follows: studies evaluating low birth weight (different classifications but with possible correspondences) and kidney function in adulthood. The evaluation of kidney function could include the glomerular filtration rate, albumin-creatinine ratio, presence of microalbuminuria, or end-stage renal disease. Subjects had to be over 18 years old, and the control group was limited to subjects with a birth weight up to the 90^th^ percentile when the article provided these data. The search included publications in English, Portuguese, French, and Spanish.

The exclusion criteria were as follows: studies involving kidney function evaluation in children, animals, or a group with a specific disease (e.g., IgA nephropathy, congenital abnormalities, minimal change nephropathy, and diabetic nephropathy); studies analyzing only kidney volume; and reviews. Furthermore, we excluded studies involving very premature births (<32 weeks), birth weights less than 1500 grams, and birth weight analyzed in quartiles; studies with missing data; and studies with different outcomes, such as the rate of low-normal creatinine clearance.

Two reviewers (JCS and MAC) independently read the titles and abstracts of the papers retrieved using the pre-defined search strategy and applied the inclusion criteria. Then, the remaining articles or the articles that did not have abstracts were obtained in full and read again, with the same eligibility criteria applied. Any selection conflicts were solved by *consensus* after discussion. Any other uncertainties were resolved by email contact with the original authors.

Then, data were extracted from the selected studies, including information on the author, date of publication, study design, number and characteristics of the participants, age, predictors, outcomes, and definitions of low birth weight, small for gestational age, IUGR, end-stage renal disease, and microalbuminuria, if applicable.

The data were analyzed using Review Manager (RevMan) 5.3. Using the Mantel-Haenszel statistical test, we compared dichotomous outcomes using the OR with the 95% CI. For continuous data, we used both the average difference (MD) and 95% CI and the statistical test of the inverse of the variance.

The heterogeneity of the data extracted from the included studies was assessed using the chi-square test and the I^2^ statistic. A difference was statistically significant if the chi-square result was *p*<0.10 and/or I^2^>50%. In such cases, we applied a random effects model. However, for the opposite result, we used a fixed effects model. In those cases, we assumed that each study estimated the same amount of effect (homogeneous) and that the results were different only due to chance.

The methodological quality of the cohort and case-control studies included in the meta-analysis was assessed according to the Newcastle-Ottawa Scale (NOS). Each study was judged from three broad perspectives: group selection, group comparability, and detection of the outcome or exposure of interest for case-control or cohort studies. As the evaluation of the NOS is currently in progress, we classified the studies as having a high risk of bias when they received between 0 and 4 stars, having a moderate risk of bias when they received between 5 and 7 stars, and having a low risk of bias when they received ≥8 stars. Cross-sectional studies were evaluated according the National Institutes of Health (NIH) Quality Assessment Tool for Observational Cohort and Cross-Sectional Studies, which comprises 14 criteria.

Sensitivity analysis was performed by excluding each study individually and performing a new statistical analysis of the remaining studies. A funnel plot was not used due to the small number of studies included in each outcome.

## RESULTS

### Search results

**Figure 1:**
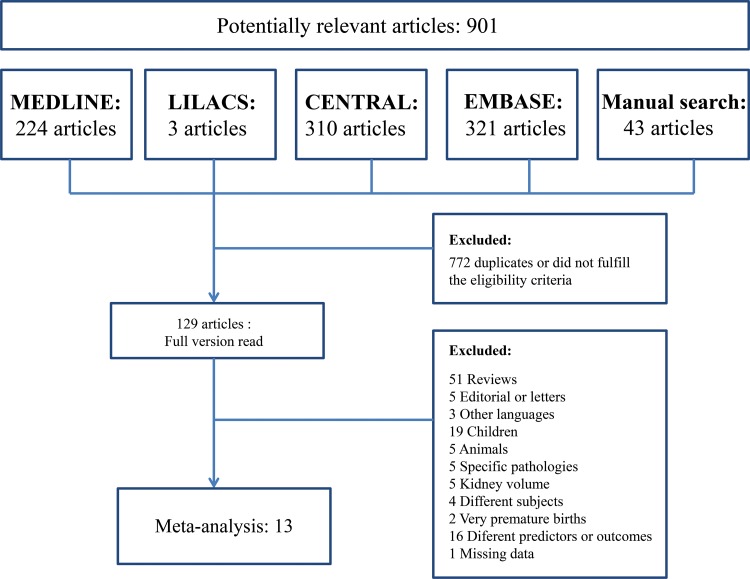
Search flowchart.

The search identified 901 articles, 858 of which were found through four electronic databases (MEDLINE: 224, LILACS: 3, CENTRAL: 310, EMBASE: 321) and 43 of which were found by manual search. After duplicates were removed and articles were deleted based on titles and abstracts, 129 articles remained. The full versions of these articles were evaluated. A total of 116 articles were excluded for several reasons (Figure 1). Finally, 13 studies were considered eligible for the systematic review and were included in the meta-analysis.

### Included studies

A total of 100466 subjects who had low birth weight or were small for gestational age were compared with 914602 controls with respect to the end-stage renal disease outcome. Fan et al. 18, Dyck et al. 19, Lackland et al. 20, and Al Salmi et al. 21,22 defined low birth weight as below 2500 g, while Ruggajo et al. 23 included subjects who were small for gestational age, defined as a birth weight less than the sex-specific 10^th^ percentile (2870 g for males and 2800 g for females). The control group included individuals whose birth weight was greater than the 10^th^ percentile but lower than the 90^th^ percentile (i.e., 3999 g). Neither Ruggajo et al. 23 nor Al Salmi et al. 21,22 mentioned an upper limit.

To evaluate microalbuminuria, we compared 1956 subjects who had low birth weight or were small for gestational age with 10125 controls. Only Laganovic et al. 24 defined the predictor as small for gestational age (birth weight <10^th^ percentile, mean: 2230 g), while Li et al. 25 and Nelson et al. 26 used low birth weight <2500 g. For the outcome, Laganovic et al. 24 defined microalbuminuria as an albumin/creatinine ratio (ACR) between 2.2 and 22 mg/mmol, while Li et al. 25 and Nelson et al. 26 defined it an ACR ≥30 mg/g. The upper limit of birth weight for the controls in the study by Nelson et al. (26) was 4500 g. Other studies, such as those by Gielen et al. 27, Laganovic et al. 24, Kistner et al. 28, Vásárhelyi et al. 29, and Hoy et al. 7, did not specify a maximum.

For the quantitative outcome, the ACRs of 255 low birth weight or small for gestational age individuals were compared with those of 312 controls. Only Laganovic et al. 24 defined the predictor as small for gestational age; Vásárhelyi et al. 29 and Hoy et al. 7 used low birth weight.

Finally, to analyze the glomerular filtration rate (GFR) (ml/min), 490 low birth weight or small for gestational age individuals were compared with 893 normal birth weight or adequate for gestational age individuals. For the analysis of GFR (ml/min/1.73 m^2^), 322 cases were compared with 366 controls. The formulas selected by each author to calculate the GFR were as follows: Gielen et al. 27 used creatinine clearance, corrected for body surface area; Laganovic et al. 24 used the Cockcroft-Gault equation; Kistner et al. 28 used the clearance of iohexol, corrected for body surface area; and Vásárhelyi et al. 29 used the Schwartz formula. Al Salmi et al. 22 calculated the GFR using serum creatinine and lean body mass.

For more information on the studies included in the meta-analysis (Table 1).

Most of the cohort and case-control studies were classified as having a moderate risk of bias according to the NOS, and the cross-sectional studies presented a mean score (which indicates the internal validity) of 52% (Tables 2 and 3).

### Summary of Results

#### End-Stage Renal Disease

**Figure 2:**
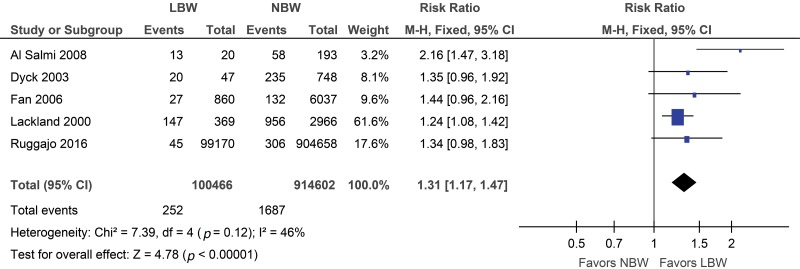
Forest plot for end-stage renal disease (ESRD).

Considering the 5 studies evaluating end-stage renal disease included in this analysis, increased risk was observed in the low birth weight group (risk ratio (RR) 1.31, 95% confidence interval (CI): 1.17, 1.47) (Figure 2). The sensitivity analysis, in which each study was excluded individually, did not change the result.

#### Glomerular filtration rate

**Figure 3:**
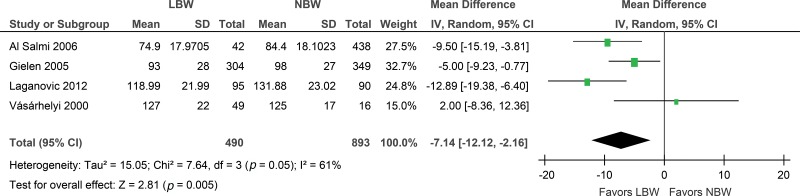
Forest plot of glomerular filtration rate (ml/min).

**Figure 4:**
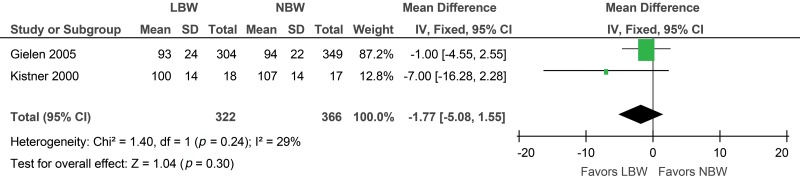
Forest plot of glomerular filtration rate (ml/min/1.73 m^2^).

An increased risk of a lower GFR (ml/min) was observed in the low birth weight group (MD -7.14; 95% CI: -12.12, -2.16). However, no statistically significant difference in the GFR (ml/min/1.73 m^2^) was found between the cases and controls (MD -1.77; 95% CI: -5.08, 1.55). The sensitivity analysis also presented the same positive results with respect to the GFR (ml/min), but when the Al Salmi et al. study was excluded, the results became insignificant (MD -6.06; 95% CI: -13.14, 1.03) (Figures 3 and 4).

#### Microalbuminuria

**Figure 5:**
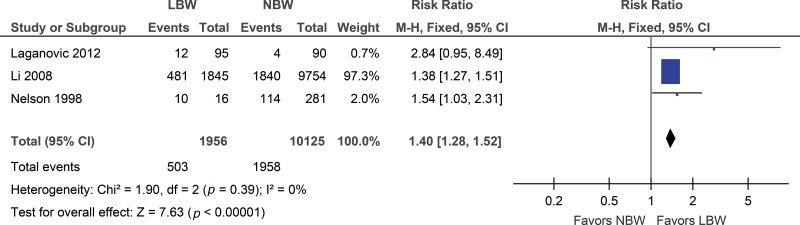
Forest plot of microalbuminuria.

Considering the 3 included studies that used microalbuminuria as an outcome, an increased risk of microalbuminuria was identified in the low birth weight group (RR 1.40; 95% CI: 1.28, 1.52) (Figure 5). The sensitivity analysis did not change the result.

#### Albumin/Creatinine ratio

**Figure 6:**
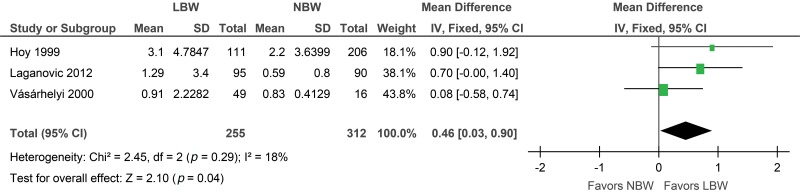
Forest plot of albumin/creatinine ratio (ACR).

A small increase in the albumin-creatinine ratio (mg/mmol) was identified in the low birth weight group compared with the controls (MD 0.46; 95% CI: 0.03, 0.90) based on the 3 relevant included studies. According to the sensitivity analysis, when either Hoy et al. 7 or Laganovic et al. 24 was excluded, the result was no longer statistically significant (Figure 6).

### DISCUSSION

According to our review, having a low birth weight or being small for gestational age during intrauterine development interferes with renal function in adulthood. We observed that these conditions are related to the development of microalbuminuria (RR 1.40; 95% CI: 1.28, 1.52) and end-stage renal disease (RR 1.31, 95% CI: 1.17, 1.47), a decreased glomerular filtration rate (ml/min) (MD -7.14; 95% CI: -12.12, -2.16), and a slightly increased albumin-creatinine ratio (mg/mmol) (MD 0.46; 95% CI: 0.03, 0.90).

The decrease in renal function likely occurs because the reduction in the nephron number in IUGR fetuses is not completely compensated for during the postnatal period 4,5. Therefore, the remaining nephrons are overloaded with increased glomerular filtration, compensatory hypertrophy and progressive fibrosis in a cycle of progressive loss of functioning units 6,7. Hinchliffe et al. 4 and Giapros et al. 5 demonstrated diminished nephron numbers and kidney lengths in this population. Indeed, the fetal response to low intrauterine oxygenation may be favorable in the short term, directing blood flow to vital organs to minimize severe hypoxia. However, vasoconstriction in peripheral organs increases risks later in life 30. Doro et al. 31 recently demonstrated that in growth-restricted fetuses, renal vascular flow is impaired in the most severe cases, and this impairment is also associated with diminished amniotic fluid volume. These authors and previous authors 32-35 suggest that diminished renal flow in these fetuses may lead to a decreased glomerular count, which has also been observed in animal models 36,37. This reduction in glomerular number may not have a clinical impact in early life, but as the functional renal reserve is diminished, later renal injuries may cause more pronounced organ dysfunction.

Another possible mechanism of injury is fetal exposure to glucocorticoids, a growth inhibitory factor that may be a cause of structural and physiological alterations in blood pressure regulation 8,9,38. Glucocorticoids activate the renin-angiotensin system 12,39 and change the hypothalamic-pituitary-adrenal axis 13 as well as the vascular system, causing endothelial damage and increasing arterial tension 40, which may impact renal function later in life. Additionally, considering that renal differentiation in mice can also be affected by a low-protein maternal diet 41,42, the impact may occur even without centralization in exposed fetuses. Because adults born small for gestational age have a diminished renal reserve to manage any mechanism of renal damage in future life, any further risk factor, such as chronic hypertension and nephrotoxic drugs, may impact renal function 6,7,43.

The finding that low birth weight is associated with renal damage in adulthood indicates the need for follow-up for individuals born below the 10^th^ percentile for weight 44. Earlier identification of individuals at a high risk for renal diseases through screening facilitates the diagnosis and management of further risk factors that may increase renal damage in the future, such as asymptomatic high blood pressure and diabetes 45. In addition, the prevention of microalbuminuria and end-stage renal disease by specific kidney protection measures, such as the use of angiotensin-converting antagonists in at-risk populations, may reduce disease progression and decrease mortality 45. These protective measures are included in guidelines that limit the high-risk population to individuals with diabetes mellitus, hypertension or a first-degree relative with diabetes, hypertension or kidney disease 46,47. But this new risk group, the low birth weight population, is not included in these primary prevention recommendations.

In contrast to previous meta-analyses 8,9, we only included studies with sample populations older than 18 years of age and excluded extremely preterm births. This allowed us to reduce the number of patients with renal manifestations of undiagnosed congenital abnormalities and inherited diseases, which are frequent causes of end-stage renal disease in the first decades of life.

The initial objective of our group was to select only longitudinal prospective cohorts; however, the literature is limited, and no results from longitudinal prospective cohorts are available. Consequently, our study was limited to cross-sectional, case-control studies and retrospective cohorts. Another important difference of our study *versus* previous studies was the exclusion of high birth weight subjects among the controls when possible, which allowed better interpretation of the results because of the U-shaped relation between birth weight (low or high) and increased urinary albumin discharge due to diabetic end-stage renal disease 17,24.

The selection of specific minorities to establish the study participants is an important source of bias in the literature and hinders the reproducibility of the results in the general population because low birth weight is suggested to be more common in some specific communities 17. Furthermore, end-stage renal disease is also related to other conditions, such as type 2 diabetes, and separating the role of the primary disease and the role of birth weight in its development is difficult. Despite all these possible sources of heterogeneity, the sensitivity analysis excluding each study individually led to non-significant changes in the results.

To study the effects of birth weight on the incidence of decreased renal function and kidney disease later in life, larger cohorts with longer follow-ups are recommended. Currently, considering the finding that renal function is impaired in adults born small for gestational age, societies must observe this population closely to determine a better follow-up strategy to reduce renal damage.

In conclusion, a positive correlation exists between low birth weight or small for gestational age and renal dysfunction in adulthood. This study provides the best evidence for this correlation based on the most homogeneous adult population available. However, well-designed prospective cohorts are needed to better understand the causality of this association and to identify possible interventions that will allow better recuperation of kidney glomerular capacity after intrauterine injury.

## AUTHOR CONTRIBUTIONS

Senra JC, Carvalho MA, Rodrigues AS, Krebs VL, Gibelli MA, Francisco RP, Bernardes LS participated in the study conception and design, collecting and synthesizing the data, and in drafting or revising the manuscript.

## Figures and Tables

**Table 1:** Studies included in the meta-analysis.

Author Year	Country	Design	Participants	Age, years	Predictor	No. of Participants	Outcomes	Frequency of Outcome/Value	OR (95% CI)
Nelson et al. (26), 1998	United States	Prospective and retrospective cohort study	Pima Indians and closely related Tohono O'odham Indians with type 2 diabetes	34±8 Range: 20 to 61	LBW <2500 g *vs.* Normal BW (between 2500 g and 4500 g)	LBW: 16 Normal BW: 281	Microalbuminuria: ACR≥30 mg/g	LBW: 10 Normal BW: 114	Microalbuminuria: 1.54 [1.03, 2.31]
Hoy et al. (7), 1999	Australia	Cross-sectional study	Aborigines in the Australian Northern Territory	27.1 Range: 20 to 38	LBW (<2500 g) *vs.* Normal BW (≥250 0 g)	LBW: 111 Normal BW: 206	Albumin/ creatinine ratio (g/mol)	LBW: 3.1 (2.2-4.3) Normal BW: 2.2 (1.7-2.8)	ACR: 0.90 [-0.12, 1.92]
Kistner et al. (28), 2000	Sweden	Retrospective cohort study	Women born full term in Stockholm	26±1.9	(1) BW <2600 g and SGA (<25^th^ percentile for gestational age) *vs.* (2) Normal BW for gestational age	(1): 18 (2): 17	Glomerular filtration rate (clearance of iohexol)	(1): 100±14 ml/min/1.73 m^2^ (2): 107±14 ml/min/1.73 m^2^	GFR (ml/min/1.73 m^2^): -7.00 [-16.28, 2.28]
Lackland (20), 2000	United States	Case-control study	Patients with chronic renal failure on dialysis from 1991 through 1996	34	LBW (<2500 g) *vs.* 2500 g ≤ BW < 3999 g	LBW: 369 Normal BW: 2966	End-stage renal disease	LBW: 147 Normal BW: 959	ESRD: 1.23 [1.08, 1.41]
Vásárhelyi et al. (29), 2000	Hungary	Prospective cohort study	Healthy young Caucasian men	Range: 19 to 21	LBW (<2500 g) *vs.* Normal BW (>2500 g)	LBW: 49 Normal BW: 16	Glomerular filtration rate (Schwartz formula) Albumin/ creatinine ratio (g/mol)	LBW: 127±22 ml/min Normal BW: 125±17 ml/min LBW: 0.91±2 g/mol Normal BW: 0.83±0.41 g/mol	GFR (ml/min): 2.00 [-8.36, 12.36] ACR: 0.08 [-0.58, 0.74]
Dyck et al. (19), 2003	Canada	Case-control study	Saskatchewan patients born after 1949 with ESRD diagnosed from 1981 through 1998	-	LBW (≥2500 g) *vs.* Normal BW (between 2500 g and 4000 g)	LBW: 47 Normal BW: 748	End-stage renal disease (dialysis or renal transplant)	LBW: 20 Normal BW: 235	ESRD: 1.35 [0.96, 1.92]
Gielen et al. (27), 2005	Belgium	Cross-sectional study	Monozygotic and dizygotic twins	25.6	LBW (<2500 g) *vs.* High BW (≥2500 g)	LBW: 304 High BW: 349	Glomerular filtration rate (creatinine clearance corrected for body surface area)	LBW: 93±28 ml/min High BW: 98±27 ml/min LBW: 93±24 ml/min/1.73 m^2^ High BW: 94±22 ml/min/1.73 m^2^	GFR (ml/min): -5.00 [-9.23, -0.77] GFR (ml/min/1.73 m^2^): -1.00 [-4.55, 2.55]
Al Salmi et al. (22), 2006	Australia	Cross-sectional study	Ausdiab study population	≥25	LBW (<2500 g) *vs.* Normal BW (≥2500 g)	LBW: 42 Normal BW: 438	Glomerular filtration rate (serum creatinine and lean body mass)	LBW: 74.9 ml/min (69.3-80.5) Normal BW: 84.4 ml/min (82.7-86.1)	GFR (ml/min): -9.50 (-15.19; -3.81)
Fan et al. (18), 2006	United States	Cross-sectional study	Caucasian and African American adults enrolled in the Medicaid program from 1993 to 1996 and diagnosed with diabetes or hypertension	Range: 18 to 50 Cases: 33.9±0.1 Controls: 37.6±0.6	LBW (<2500 g) *vs.* Normal BW (between 2500 g and 3999 g)	LBW: 860 Normal BW: 6037	End-stage renal disease	LBW: 27 Normal BW: 132	ESRD: 1.44 [0.96, 2.16]
Al Salmi et al. (21), 2008	Australia	Case-control study	Patients attending the Nephrology Department during the first quarter of 2004	Cases: 63.1±14.4 Controls: 62.7±16.6	LBW (<2500 g) *vs.* Normal BW	LBW: 20 Normal BW: 193	End-stage renal disease (GFR>15 ml/min/1.73 m^2^ or on dialysis therapy)	LBW: 13 Normal BW: 58	ESRD: 2.16 [1.47, 3.18]
Li et al. (25), 2008	United States	Cross-sectional study	Patients with diabetes or hypertension or with a first-degree relative affected, registered in KEEP	49.1±13.5 Range: 18 to 75	LBW (<2500 g) *vs.* Normal BW (between 2500 g and 3999 g)	LBW: 1845 Normal BW: 9754	Microalbuminuria: ACR≥30 mg/g	LBW: 481 Normal BW: 1840	Microalbuminuria: 1.38 [1.27, 1.51]
Laganovic et al. (24), 2012	Croatia	Cross-sectional study	Healthy men	Cases: 21±0.89 Controls: 21.5±1.02	SGA (<10^th^ percentile for gestational age according to the Croatian population) *vs.* AGA (>2500 g and full term)	SGA: 95 AGA: 90	Glomerular filtration rate (Cockcroft-Gault equation) Microalbuminuria: 2.2<ACR<22 mg/mmol Albumin/ creatinine ratio (g/mol)	SGA: 118.99±21.99 ml/min AGA: 131.88±23.02 ml/min SGA: 12 AGA: 4 SGA: 1.29±3.4 g/mol AGA: 0.59±0.8 g/mol	GFR (ml/min): -12.89 [-19.38, -6.40] Microalbuminuria: 2.84 [0.95, 8.49] ACR: 0.70 [-0.00, 1.40]
Ruggajo et al.(23), 2016	Norway	Retrospective cohort study	Subjects registered in the Norwegian Renal Registry with ESRD or with a sibling who had ESRD or was SGA	Range: 18 to 42	LBW (< the sex-specific 10^th^ percentile) *vs*. Normal BW (> 10th percentile)	LBW: 99170 Normal BW: 904964	End-stage renal disease (dialysis or kidney transplantation)	LBW: 45 Normal BW: 306	ESRD: 1.34 [0.98, 1.83]

ACR, albumin/creatinine ratio; AGA, adequate for gestational age; BW, birth weight; ESRD, end-stage renal disease; GFR, glomerular filtration rate; LBW, low birth weight; SGA, small for gestational age.

**Table 2:** Newcastle-Ottawa Quality Assessment Scale of the included studies (cohorts and case-controls).

Study	Representativeness of the exposed cohort // Is the case definition adequate?	Selection of the non-exposed cohort // Representativeness of the cases	Ascertainment of exposure//Selection of controls	Demonstration that the outcome of interest was not present at the start of the study // Definition of controls	Comparability of (cohorts/cases and controls) based on the design or analysis	Assessment of (outcome/exposure)	Was the follow-up long enough for outcomes to occur? // Same method of ascertainment for cases and controls	Adequacy of the follow-up of cohorts // Non-response rate	Score
Nelson et al. (26), 1998	*	*	*	*	*	*		*	**7**
Kistner et al. (28), 2000	*	*	*	*	*			*	**6**
Lackland et al. (20) 2000	*	*	*		**	*	*		**7**
Vásárhelyi et al. (29), 2000	*		*	*	*	*			**5**
Dyck et al. (19), 2003	*	*	*		**	*	*		**7**
Al Salmi et al. (22), 2008	*	*	*		*				**4**
Ruggajo et al. (23), 2016	*	*	*				*	*	**5**

**Table 3:** Quality Assessment Tool for Cross-Sectional Studies included in the meta-analysis.

Study	Research objective clearly stated?	Study population clearly defined?	Participation rate of eligible persons at least 50%?	All subjects recruited from similar populations?	Justification of the sample size?	Exposure measured prior to outcome?	Sufficient time frame to see an association between exposure and outcome?	Different levels of exposure measured?	Exposure measures clearly defined?	Exposure assessed more than once over time?	Outcome measures clearly defined?	Outcome assessors blinded to exposure status?	Loss to follow-up after baseline ≤20%?	Key potential confounders statistically adjusted for?	Score
Hoy et al. (7), 1999	**Y**	**Y**	**NR**	**Y**	**N**	**N**	**N**	**N**	**Y**	**N**	**Y**	**NR**	**N**	**N**	**5**
Gielen et al. (27), 2005	**Y**	**Y**	**Y**	**Y**	**N**	**Y**	**N**	**N**	**Y**	**N**	**Y**	**NR**	**N**	**Y**	**8**
Al Salmi et al. (22) 2006	**Y**	**Y**	**Y**	**Y**	**N**	**Y**	**CD**	**N**	**Y**	**N**	**Y**	**NR**	**N**	**Y**	**8**
Fan et al. (18) 2006	**Y**	**Y**	**Y**	**Y**	**N**	**Y**	**N**	**N**	**Y**	**N**	**Y**	**NR**	**N**	**Y**	**8**
Li et al. (25) 2008	**Y**	**Y**	**Y**	**Y**	**N**	**Y**	**Y**	**N**	**Y**	**Y**	**Y**	**NR**	**N**	**Y**	**10**
Laganovic et al. (24) 2012	**Y**	**N**	**Y**	**NR**	**N**	**Y**	**N**	**N**	**Y**	**N**	**Y**	**NR**	**N**	**N**	**5**

CD, cannot determine; N, no; NR, not reported; Y, yes.
